# Impaired Excitatory Neurotransmission in the Urinary Bladder from the Obese Zucker Rat: Role of Cannabinoid Receptors

**DOI:** 10.1371/journal.pone.0157424

**Published:** 2016-06-10

**Authors:** Igor Blaha, Paz Recio, María Pilar Martínez, María Elvira López-Oliva, Ana S. F. Ribeiro, Ángel Agis-Torres, Ana Cristina Martínez, Sara Benedito, Albino García-Sacristán, Vítor S. Fernandes, Medardo Hernández

**Affiliations:** 1 Departamento de Urología, Hospital General Universitario Gregorio Marañón, 28007-Madrid; 2 Departamento de Fisiología, Facultad de Farmacia, Universidad Complutense de Madrid, 28040-Madrid; 3 Departamento de Anatomía y Anatomía Patológica Comparadas, Facultad de Veterinaria, Universidad Complutense de Madrid, 28040-Madrid; Cinvestav-IPN, MEXICO

## Abstract

Metabolic syndrome (MS) is a known risk factor for lower urinary tract symptoms. This study investigates whether functional and expression changes of cannabinoid CB_1_ and CB_2_ receptors are involved in the bladder dysfunction in an obese rat model with insulin resistance. Bladder samples from obese Zucker rat (OZR) and their respective controls lean Zucker rat (LZR) were processed for immunohistochemistry and western blot for studying the cannabinoid receptors expression. Detrusor smooth muscle (DSM) strips from LZR and OZR were also mounted in myographs for isometric force recordings. Neuronal and smooth muscle CB_1_ and CB_2_ receptor expression and the nerve fiber density was diminished in the OZR bladder. Electrical field stimulation (EFS) and acetylcholine (ACh) induced frequency- and concentration-dependent contractions of LZR and OZR DSM. ACh contractile responses were similar in LZR and OZR. EFS-elicited contractions, however, were reduced in OZR bladder. Cannabinoid receptor agonists and antagonists failed to modify the DSM basal tension in LZR and OZR In LZR bladder, EFS responses were inhibited by ACEA and SER-601, CB_1_ and CB_2_ receptor agonists, respectively, these effects being reversed by ACEA plus the CB_1_ antagonist, AM-251 or SER-601 plus the CB_2_ antagonist, AM-630. In OZR bladder, the inhibitory action of ACEA on nerve-evoked contractions was diminished, whereas that SER-601 did not change EFS responses. These results suggest that a diminished function and expression of neuronal cannabinoid CB_1_ and CB_2_ receptors, as well as a lower nerve fiber density is involved in the impaired excitatory neurotransmission of the urinary bladder from the OZR.

## Introduction

Metabolic syndrome (MS) is a cluster of metabolic disorders and risk factors of cardiovascular disease, including central obesity, type II diabetes mellitus (IIDM), dyslipidemia and hypertension which share a common insulin resistance, a compensatory hyperinsulinemia and glucose intolerance [1]. Both the MS and the IIDM are known risk factors for lower urinary tract symptoms (LUTS) [2]. In fact, the proinflammation and myopathy of the bladder induced by metabolic disorders, such as obesity and IIDM, have a key role in causing bladder dysfunction and urinary incontinence (UI) [3–6].

Endocannabinoid system, which comprises CB_1_ and CB_2_ cannabinoid receptors, endocannabinoids, such as anandamide and enzymes involved in endocannabinoid synthesis and degradation, plays a pivotal role in physiological control of micturition [7–10]. In human bladder, CB_1_ and CB_2_ cannabinoid receptors have been identified in parasympathetic nerves distributed in the detrusor smooth muscle (DSM) and urothelium, modulating the neurotransmitter release [7]. Potentiation of endocannabinoid activity depresses the Aδ and C-fiber hyperactivity of primary bladder afferents, thus reducing bladder overactivity and hypersensitivity, so endocannabinoid system has been proposed as a therapeutic target in LUTS [8–10].

Obese Zucker rat (OZR) is a well-established genetic model of insulin resistance and MS caused by a dysfunctional gene of the leptin receptor [11]. These animals were used as a model of MS because of impaired glucose tolerance associated with the inherited obesity gene mutation, and they progressively develop obesity, IIDM and hypertension [11]. The IIDM, obesity and complications arising therefrom alter the normal function of the lower urinary tract in OZR, which makes them an excellent model for the study of bladder dysfunction associated with MS in men. Cystometry in OZR showed a decreased voiding frequency and nonvoiding contractions, contraction pressure and threshold pressure, as well as an increased fill volume, volume voided and residual volume [12]. Moreover, in OZR, the external urethral sphincter presents fibrosis and edema of the periurethral muscularis, thus producing a marked disruption of the striated muscular structure. In the OZR bladder wall is also characteristic the presence of edema, vasculopathy and an altered ion channels expression in detrusor smooth muscle cells [12]. Although obesity and diabetes are risk factors for UI [3–6], there is little definitive data on the pathophysiological mechanisms responsible for bladder dysfunction in MS. Therefore, the aim of the current study was to investigate whether functional and expression changes of cannabinoid receptors, which have been identified in parasympathetic nerves distributed in the DSM [7], may modify the nerve-evoked contractions of the urinary bladder from OZR.

## Materials and Methods

### Animal model

Male OZR (fa/fa, n = 20) and their control counterpart, lean Zucker rat (LZR) (fa/-, n = 20), were purchased from Charles River Laboratories (Barcelona, Spain) at 8–10 weeks of age and maintained on standard chow and water ad libitum until they were sacrificed at 17–18 weeks. The experiments were approved by the Animal Experimentation Ethics Committee of Complutense University and conformed to the US National Institutes of Health Guidelines for the Care and the Use of Laboratory Animals.

### Isolation of bladder samples

Animals were killed by cervical dislocation and exsanguination. Next, the bladder was quickly removed and placed in a cold physiological saline solution (PSS) at 4°C. The composition of PSS was (in mM): NaCl 119, KCl 4.6, MgCl_2_ 1.2, NaHCO_3_ 24.9, glucose 11, CaCl_2_ 1.5, KH_2_PO_4_ 1.2, EDTA (ethylenediamine tetraacetic acid) 0.027. The solution was maintained at 37°C and continuously gassed with 95% O_2_ and 5% CO_2_ to maintain pH at 7.4. The adjacent connective and fatty tissues were removed with care and longitudinal strips were obtained from the LZR and OZR DSM.

### Western blot

Dome samples of urinary bladder, of the same size as those used for isometric force recording, were homogenized in lyses buffer containing 10 mM Tris-HCl (pH 7.4), 1% SDS, 1 mM sodium vanadate and 0.01% protease inhibitor cocktail (all from Sigma Aldrich, Madrid, Spain) and centrifugated for 15 min at 14000 g at 4°C. Equal amounts of protein extract (20 μg) (DC Protein Assay Kit, Bio-Rad, Madrid, Spain) from control LZR and OZR group samples were separated in a 10% polyacrylamide gel (SDS-PAGE) and, after migration, were transferred to a polyvinylidene fluoride (PVDF) membrane (GE Healthcare, Madrid, Spain). All membranes were blocked by 5% non-fat dry milk for 1 h at room temperature. For immunodetection, the membranes were incubated overnight at 4°C with the rabbit polyclonal primary antibodies: anti-CB_1_ (1/1000) (sc-20754) and anti-CB_2_ (1/1000) (sc-25494) (Santa Cruz Biotechnology, Madrid, Spain). Membranes were then washed in 0.05% Tween-20, incubated with HRP-conjugated secondary antibodies for 1 h at room temperature, and then washed and visualized by chemiluminescence (ECL Select-kit, GE Healthcare, Madrid, Spain) using anti-β-actin antibody (1/20000) (Santa Cruz Biotechnology, Madrid, Spain) as the loading control.

### Immunohistochemistry

Detrusor smooth muscle (DSM) samples were fixed in 4% paraformaldehyde in 0.1 M phosphate buffer, pH 7.4 (PB), for 2 to 4 h at 4°C, and subsequently placed in 30% sucrose in 0.1 M PB for cryoprotection. The tissue was embedded and frozen in OCT compound (Sakura Finetek, Europe B.V.), and stored at -80°C. Transversal sections 5 μm thick were obtained by means of a cryostat and preincubated in 10% normal goat serum in PB containing 0.3% Triton-X-100, for 2–3 h. Then, sections were incubated with either rabbit anti-CB_1_ or anti-CB_2_ receptor antibodies diluted 1:50 plus a mouse anti-protein gene product 9.5 (anti-PGP 9.5), as neuronal marker, diluted 1:20 during 48 h at 4°C, washed and reacted with the second antibodies Alexa Fluor 594 goat-antirabbit (1:200 dilution) to detect CB_1_ and CB_2_ and Alexa Fluor 488 goat-antimouse (1:200 dilution) to detect PGP 9.5 for 2 h at room temperature. The slides were covered with a specific mounting medium with DAPI (Invitrogen) which stains all cell nuclei.

The intensity of immunostaining for each antibody was measured using ImageJ free software (National Institutes of Health USA). We have used ImageJ on the original raw images to clearly show the levels of immunostaining for each protein. All photographs were obtained in the same conditions.

### Myographs for isometric force recordings

Urothelium-intact DSM strips were dissected to a size of 4 mm long and 2 mm wide, and suspended horizontally, between two parallel L-shaped stainless steel wires, with one end connected to an isometric force transducer (Grass FT03C) and the other to a micrometer screw, in 5 ml organ baths and Mulvany myographs containing PSS at 37°C continuously gassed with carbogen (95% O_2_ and 5% CO_2_) to obtain a final pH of 7.4. The signal was continuously displayed and recorded using a data acquisition system PowerLab hardware and Chart v5.3 software (DMT, Aarhus, Denmark). Active tension of 1.2 g was applied to the strips and they were allowed to equilibrate for 60 min.

The contractile ability of the strips was determined by exposing them to 124 mM potassium rich PSS (KPSS). In order to block the DSM relaxation mediated via activation of beta-adrenergic receptors and nitric oxide (NO), strips were incubated with propranolol (10 μM) and N^G^-nitro-L-arginine (L-Noarg, 100 μM), beta-adrenergic receptor and NO synthase, respectively, inhibitors for 30 min, and these drugs were present throughout the experiment. Under these conditions on basal tension of DSM preparations, EFS was performed by delivering rectangular pulses (1 ms duration, 0.5–16 Hz, 20 s trains, with constant current output adjusted to 75 mA), at 4 min intervals, from a Cibertec CS20 stimulator (Barcelona, Spain). A first control response curve to EFS and acetylcholine (ACh, 10 nM-1 mM) was obtained on the same DSM strip from both LZR and OZR. The bath solution was then changed every 15 min for a period of 90 min. The preparations were incubated primarily with the CB_1_ and CB_2_ cannabinoid receptor agonists for 10 min and then a second contraction curve was constructed. Afterwards, strips were washed out and treated with the specific treatments for 30 min, and a third contraction curve was constructed. Control curves to EFS, ACh carbachol (CCh) or α,β-methylene-adenosine 5'-triphosphate (α,β-Met-ATP) were run in parallel throughout the experiment.

### Drugs

The following drugs were used: acetylcholine (ACh), atropine, carbachol (CCh), N^G^-nitro-L-arginine (L-Noarg) and tetrodotoxin (TTX), all from Sigma (USA). α,β-methylene-adenosine 5'-triphosphate (α,β-Met-ATP), arachidonyl-2'-chloroethyl amide (ACEA), N-(piperidin-1-yl)-5-(4-iodophenyl)-1-(2,4-dichlorophenyl)-4-methyl-1H-pyrazole-3-carboxamide (AM-251), 6-iodo-2-methyl-1-[2-(4-morpholinyl)ethyl]-1H-indol-3-yl](4-methoxyphenyl)methanone (AM-630) and N-(adamant-1-yl)-6-isopropyl-4-oxo-1-pentyl-1,4-dihydroquinoline-3-carboxamide (SER-601) from Tocris (UK). ACEA was dissolved in 96% ethanol. AM-251, AM-630 and SER-601 were dissolved in 10% dimethylsulphoxide (DMSO). The other drugs were dissolved in distilled water. The solvents, at the final concentration (< 0.05%) used in the bath, had no effect on the contractility of the detrusor preparations. The primary CB_1_ and CB_2_ antibodies were from Santa Cruz Biotechnology (Madrid, Spain) and PGP 9.5 was from Abcam (Cambridge, UK). The secondary antibodies were from Invitrogen (Paisley, UK).

### Calculations and Statistics

Sensitivity to ACh, CCh and α,β-Met-ATP is expressed in terms of pD_2_, where pD_2_ = -log EC_50_ and EC_50_ is the agonist concentration needed to produce half-maximal response. pD_2_ was estimated by computerized non-linear regression analysis (GraphPad Prism, USA). Results are shown as the percentage of the KPSS-induced contraction and represent the mean±SEM of *n* (number of preparations, 2 strips were isolated from each bladder). Differences were analyzed by paired and unpaired Student´s *t*-test for comparison between two groups and by one-way analysis of variance (ANOVA) followed by Bonferroni´s *post hoc* test for multiple comparisons. The differences were considered significant with a probability level of *P*<0.05.

## Results

### General parameters

At 17–18 weeks of age, OZR showed a significant increase in body weight (489±7 g* versus 363±5 g) and blood glucose after non-fasting (143±12 mg/ml* versus 101±6 mg/ml) (*P<0.05 compared with LZR value, unpaired Student´s *t*-test, n = 20 from 20 LZR and OZR). Insulin, cholesterol and triglycerides plasma concentrations were also increased in OZR (1.10±0.20 ng/ml and 4.30±0.50 ng/ml*, 0.92±0.04 mg/ml and 1.83±0.13 mg/ml* and 0.99±0.13 mg/ml and 3.34±0.33 mg/ml*, for insulin, cholesterol and triglycerides in LZR and OZR, respectively, *P<0.05 versus LZR value, unpaired Student´s *t*-test, n = 20 from 20 LZR and OZR). Therefore, OZR were significantly heavier than the LZR and exhibited mild hyperglycaemia, hyperinsulinemia and dyslipidemia with elevated total cholesterol and triglyceride levels. Systolic blood pressure was similar in LZR and OZR (123±3 and 129±6 mm Hg, respectively, n = 20 from 20 LZR and OZR).

### Cannabinoid receptor expression and nerve fiber density in bladder from LZR and OZR

**Fig 1:**
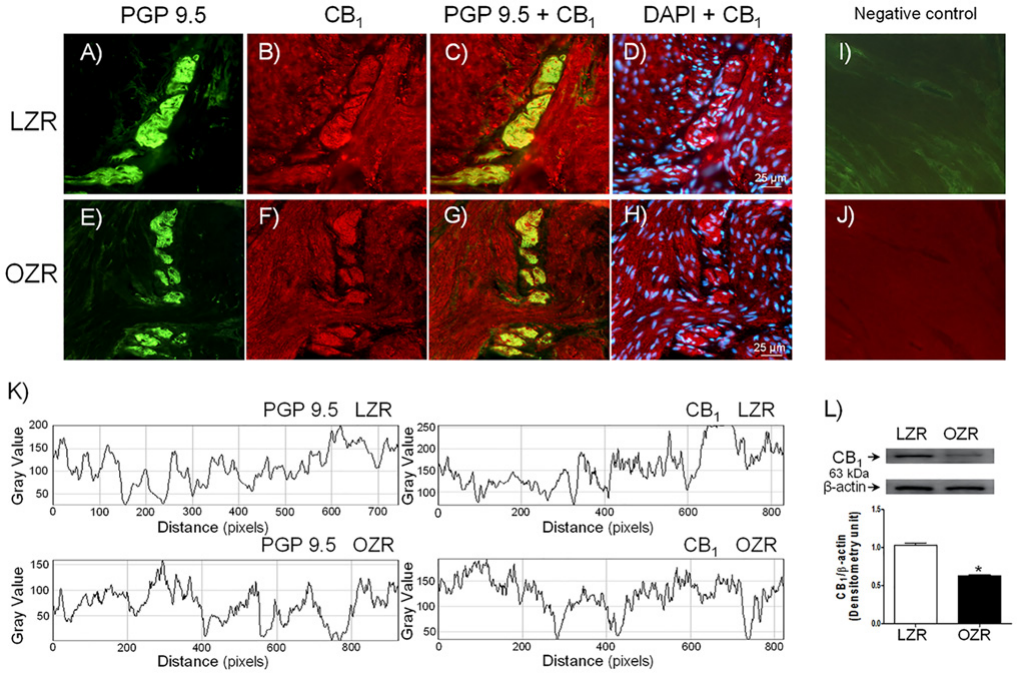
CB_1_ receptor expression and the nerve fiber density are diminished in bladder strips from OZR. CB_1_ cannabinoid receptor expression in representative detrusor muscle transverse sections from a total of 5 lean (LZR) and obese (OZR) Zucker rat bladders. Detrusor overall innervation was visualized using the general nerve marker PGP 9.5 (green areas) (A and E). Bladder CB_1_ receptor immunofluorescence from LZR (B) and OZR (F) reveals immunopositive nerve trunks (red areas), running parallel to the smooth muscle bundles. Same fields (A, B, E and F). Immunofluorescence double labelling for PGP 9.5 and CB_1_ receptor in the smooth muscle, demonstrate neuronal co-localization (yellow areas) (C and G). Cell nuclei were counterstained with DAPI (blue areas) (D and H). Scale bars indicate 25 μm. Negative controls showing the lack of immunoreactivity in sections incubated in the absence of the primary antibody (I and J). Comparison of fluorescence density of nerve fibers and CB_1_ receptors in LZR and OZR, by using ImageJ, showing a diminished fluorescence intensity of nerve fibers and CB_1_ receptors in OZR bladder (K). Western blot of detrusor smooth muscle membranes from LZR and OZR incubated with CB_1_ antibody showing a 63 kDa major band, which corresponded to the expected molecular weight, suggesting a reduced CB_1_ protein expression in OZR bladder (L). *P<0.05 vs control value, unpaired Student´s *t-*test.

**Fig 2:**
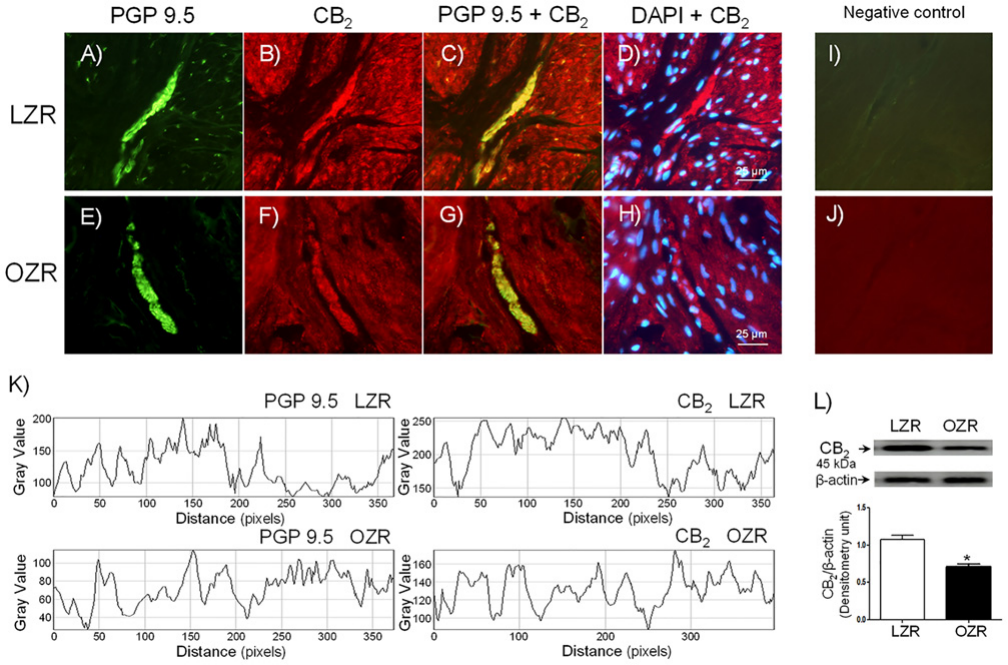
CB_2_ receptor expression and the nerve fiber density are diminished in bladder strips from OZR. CB_2_ cannabinoid receptor expression in representative detrusor muscle transverse sections from a total of 5 lean (LZR) and obese (OZR) Zucker rat bladders. Detrusor overall innervation was visualized using the general nerve marker PGP 9.5 (green areas) (A and E). Bladder CB_2_ receptor immunofluorescence from LZR (B) and OZR (F) reveals immunopositive nerve fibers (red areas), running parallel to the smooth muscle bundles. Same fields (A, B, E and F). Immunofluorescence double labelling for PGP 9.5 and CB_2_ receptor in the smooth muscle, demonstrate neuronal co-localization (yellow areas) (C and G). Cell nuclei were counterstained with DAPI (blue areas) (D and H). Scale bars indicate 25 μm. Negative controls showing the lack of immunoreactivity in sections incubated in the absence of the primary antibody (I and J). Comparison of fluorescence density of nerve fibers and CB_2_ receptors in LZR and OZR, by using ImageJ, showing a diminished fluorescence intensity of nerve fibers and CB_2_ receptors in OZR bladder (K). Western blot of detrusor smooth muscle membranes from LZR and OZR incubated with CB_2_ antibody showing a 45 kDa major band, which corresponded to the expected molecular weight, suggesting a reduced CB_2_ protein expression in OZR bladder (L). *P<0.05 vs control value, unpaired Student´s *t-*test.

CB_1_ and CB_2_ receptor expression was investigated by using CB_1_ and CB_2_ selective antibodies combined with the neuronal marker PGP 9.5. CB_1_ (Fig 1) and CB_2_ (Fig 2) receptor expression was observed within nerve fibers distributed among smooth muscle bundles, as well as in the detrusor layer from LZR (Figs 1A–1D and 2A–2D) (n = 5 from 5 rats) and OZR (Figs 1E–1H and 2E–2H) (n = 5 from 5 rats). CB_1_ (Fig 1F and 1G) and CB_2_ (Fig 2F and 2G) cannabinoid receptor protein expression was reduced in nerves and smooth muscle of bladder from OZR. PGP 9.5 also showed a lower bladder nerve fiber density in OZR (Figs 1A–1E and 2A–2E). No immunoreactivity could be detected in sections incubated in the absence of the corresponding primary antisera (Figs 1I, 1J and 2I, 2J). By using of ImageJ, a minor fluorescence intensity of nerve fibers (Figs 1K and 2K), as well as of CB_1_ (Fig 1K) and CB_2_ (Fig 2K) cannabinoid receptors was detected in bladder from OZR. By western blot, CB_1_ (Fig 1L) and CB_2_ (Fig 2L) receptor antibodies recognized bands of approximately 63 kDa and 45 kDa, respectively, which corresponded to the expected molecular weight, indicating a CB_1_ and CB_2_ protein expression in bladder membranes from LZR (n = 6 from 6 rats) and OZR (n = 6 from 6 rats). The expression of both cannabinoid receptor subtype protein was reduced in OZR bladder (Figs 1L and 2L).

### Functional studies

DSM strips from LZR (n = 20 from 10 rats) and OZR (n = 20 from 10 rats) were allowed to equilibrate to a passive tension of 0.9±0.2 g. Under these conditions, KPSS (124 mM) produced similar contractions in DSM from LZR and OZR (3.9 ± 0.5 g and 3.7 ± 0.3 g in control and obese rats, respectively).

#### Contractile responses to ACh, CCh, α,β-Met-ATP and EFS

Concentration- and frequency-response contraction curves to ACh, CCh and α,β-Met-ATP and EFS from LZR and OZR, on preparation basal tension, were constructed to assess changes of bladder excitatory neurotransmission from OZR.

**Fig 3:**
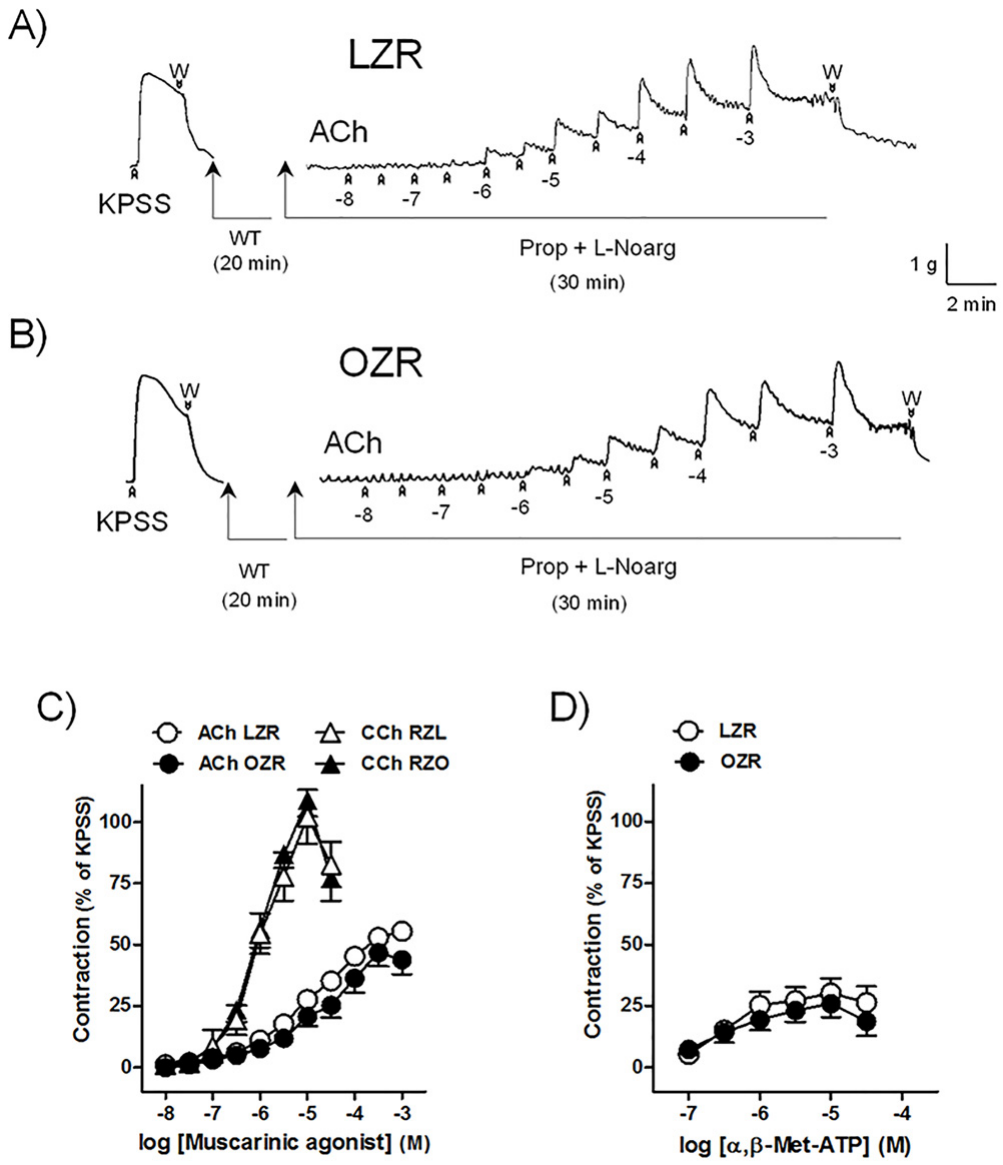
Contractile responses to ACh, CCh and α,β-Met-ATP are similar in LZR and OZR bladder strips. Isometric force recordings showing the contractions evoked by potassium rich (124 mM) physiological saline solution (KPSS) and acetylcholine (ACh, 10 nM-1 mM), on the basal tone, of lean (LZR) (A) and obese (OZR) (B) Zucker rat detrusor strips, treated with propranolol (Prop,10 μM) and N^G^-nitro-L-arginine (L-Noarg, 100 μM). Vertical bar shows tension in g and horizontal bar time in min. W: wash. WT: waiting time. (C, D) Concentration-response contraction curve to ACh and carbachol (CCh, 10 nM-30 μM) (C) and α,β-Met-ATP (100 nM-30 μM) (D) in the bladder from LZR (control, open circles and triangles) and OZR (closed circles and triangles). Results are shown as mean ± SEM percent of KPSS induced contraction in 6–7 preparations from 4–6 rats.

**Fig 4:**
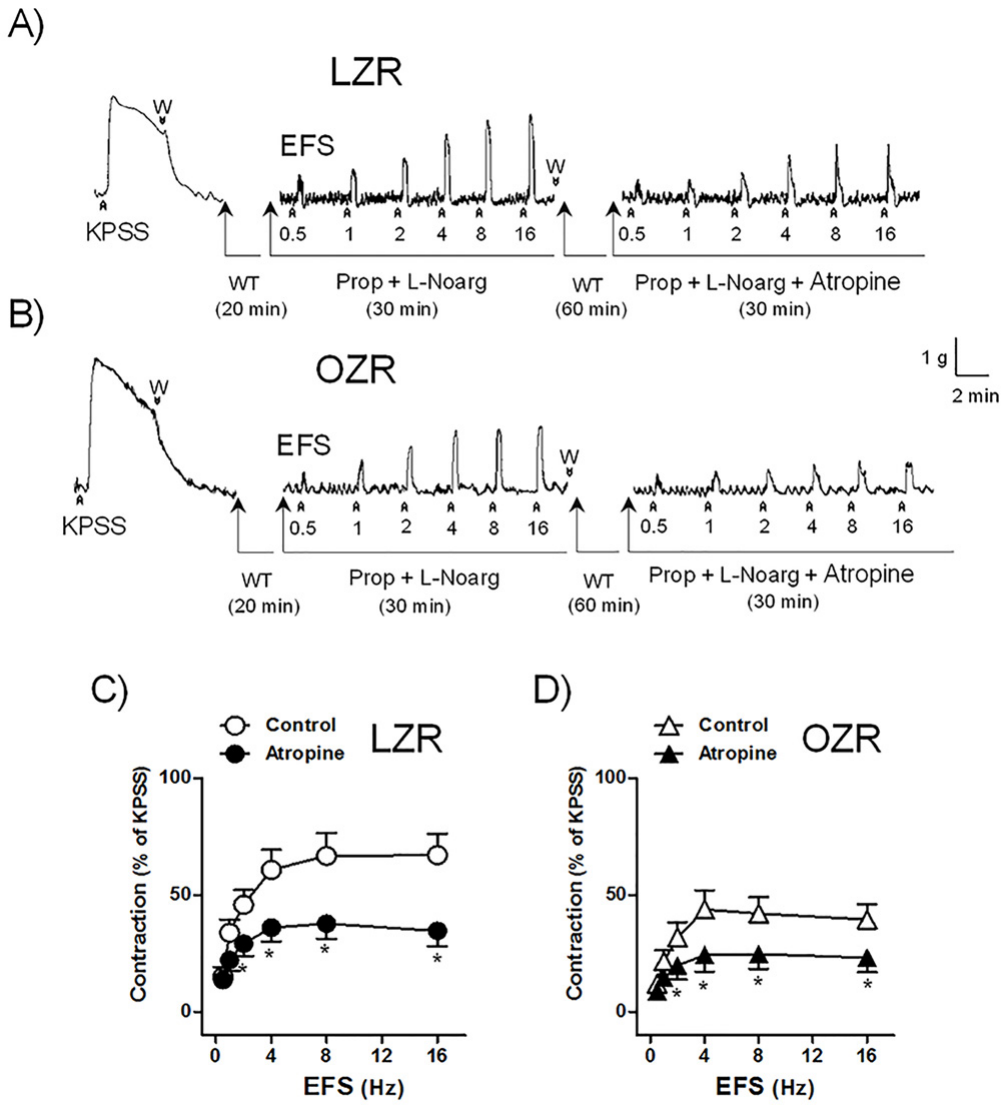
EFS-induced contractions are reduced in bladder strips from OZR. Isometric force recordings showing the contractions evoked by potassium rich (124 mM) physiological saline solution (KPSS) and electrical field stimulation (EFS, 1 ms in duration, 0.5 to 16 Hz and 20-second trains), on the basal tone, of lean (LZR) (A) and obese (OZR) (B) Zucker rat detrusor strips, treated with propranolol (Prop,10 μM) and N^G^-nitro-L-arginine (L-Noarg, 100 μM), in the absence or presence of atropine (1 μM). Vertical bar shows tension in g and horizontal bar time in min. W: wash. WT: waiting time. Frequency-response contraction curve to EFS in the detrusor from LZR (C) and OZR (D) in the absence (control, open circles and triangles) or presence (closed circles and triangles) of atropine. Results are shown as mean ± SEM percent of KPSS induced contraction in 7 preparations from 4 rats. *P<0.05 vs control value, one-way analysis of variance followed by Bonferroni method.

ACh (10 nM-1 mM), CCh (10 nM-30 μM), and α,β-Met-ATP (100 nM-30 μM) induced similar dose-dependent contractions in both LZR and OZR DSM (Table 1) (Fig 3). EFS (0.5–16 Hz) evoked frequency-dependent contractions of LZR and OZR bladders which were abolished by inhibition of neuronal voltage-gated Na^+^ channels with 1 μM TTX, thus indicating its neurogenic character. EFS contractions were diminished in OZR bladder in comparison with those obtained in LZR (maximal contraction obtained at 16 Hz of 67±9% and 39±7%* in bladders from LZR (n = 7 from 4 rats) and OZR (n = 7 from 4 rats), respectively, (*P<0.05 vs control value, one-way analysis of variance followed by Bonferroni method) (Fig 4). Blockade of muscarinic receptors with atropine caused an inhibition of 49% and 42% contraction elicited at 16 Hz stimulation frequency in bladder from LZR (n = 7 from 4 rats) and OZR (n = 7 from 4 rats), respectively (Fig 4).

**Table 1:** Contractile responses to acetylcholine (ACh), carbachol (CCh) and α,β-methylene-adenosine 5'-triphosphate (α,β-Met-ATP), in the detrusor smooth muscle from lean (LZR) and obese (OZR) Zucker rat.

		ACh			CCh			α,β-Met-ATP	
	*n*	*pD*_*2*_	*Emax* (%)	*n*	*pD*_*2*_	*Emax* (%)	*n*	*pD*_*2*_	*Emax* (%)
LZR	6	4.7±0.1	55.5±3.6	7	6.1±0.1	82.7±9.1	6	6.6±0.1	26.4±6.5
OZR	6	4.7±0.2	46.7±5.5	6	6.1±0.1	77.1±9.2	6	6.7±0.2	18.7±5.9

Results represent the mean±s.e.mean of *n* preparations from 4–6 rats. *Emax* is the maximal relaxation, expressed as a percentage of the K^+^-enriched physiological saline solution (KPSS, 124 mM)-induced contraction, obtained for each drug. pD_2_ = -log EC_50_, where EC_50_ is the concentration of agonist producing 50% of the *Emax*.

#### Effects of CB_1_ and CB_2_ cannabinoid receptor agonists and antagonists on the DSM tension

Since that CB_1_ and CB_2_ receptors are present not only in neurons but also in smooth muscle layer, we investigated if selective agonists or antagonists of cannabinoid receptors modify the DSM contractility in control and obese rats. CB_1_ and CB_2_ receptor agonists (at 10 μM) and antagonists (at 1 μM), however, failed to modify the basal DSM tension from LZR (n = 7 from 5 rats) and OZR (n = 7 from 5 rats).

#### Effects of CB_1_ and CB_2_ cannabinoid receptor agonists and antagonists on contractions to EFS

In order to study the modulation exerted by prejunctional cannabinoid receptors on excitatory neurotransmission from LZR and OZR bladders, we assessed the effect of selective CB_1_ and CB_2_ agonists and antagonists on nerve-evoked contractions.

**Fig 5:**
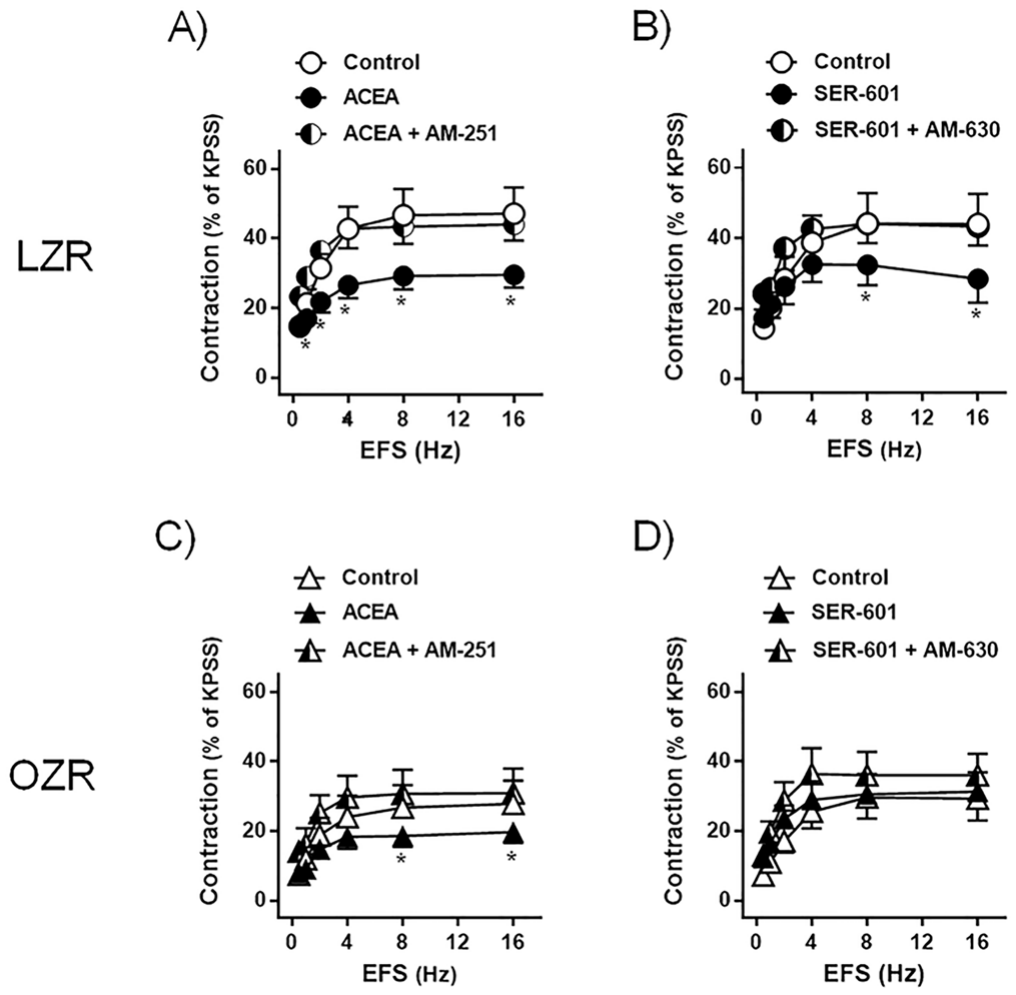
The inhibitory action of CB_1_ receptor agonists on EFS-induced contractions is diminished, whereas that CB_2_ receptor agonists failed to modify EFS-evoked responses in bladder strips from OZR. Frequency-response contraction curve to EFS in the detrusor strips from lean (LZR) (A and B) and obese (OZR) Zucker rat (C and D), treated with propranolol (10 μM) and N^G^-nitro-L-arginine (100 μM), in the absence (control, open symbols) or presence of ACEA (10 μM) and SER-601 (10 μM) (closed symbols), selective CB_1_ and CB_2_ cannabinoid receptor agonists, respectively, and ACEA plus the CB_1_ antagonist, AM-251 (1 μM), and SER-601 plus the CB_2_ receptor antagonist, AM-630 (1 μM) (half-closed symbols). Results are shown as mean ± SEM percent of KPSS induced contraction in 8–11 preparations from 5–7 rats. *P<0.05 vs control value, one-way analysis of variance followed by Bonferroni method.

In LZR bladder, ACEA (10 μM, n = 11 from 7 rats) (Fig 5A) (13) and SER-601 (10 μM, n = 8 from 5 rats) [13] (Fig 5B), selective CB_1_ and CB_2_ receptor agonists, respectively, inhibited the EFS responses, these effects being reverted by the pre-treatment with ACEA plus the CB_1_ receptor antagonist AM-251 (1 μM) [14] and SER-601 plus the CB_2_ receptor antagonist AM-630 (1 μM) [15]. In OZR bladder, the inhibitory action of ACEA on nerve-evoked contractions was reduced (n = 9 from 6 rats) (Fig 5C), whereas that SER-601 failed to modify EFS responses (n = 11 from 7 rats) (Fig 5D).

## Discussion

Our results suggest that a reduced neuronal CB_1_ and CB_2_ cannabinoid receptor function and expression, as well as a diminished nerve fiber density is involved in the impaired excitatory neurotransmission of the bladder from OZR. This conclusion is supported by the following observations: (i) Pre- and post-junctional CB_1_ and CB_2_ receptor protein expression and the nerve fiber density were diminished in OZR bladder. (ii) EFS contractions of DSM strips from OZR were reduced, whereas that ACh responses, however, were similar in LZR and OZR bladders. (iii) CB_1_ and CB_2_ receptor agonists and antagonists, did not change the DSM basal tension from LZR and OZR. In LZR bladder, EFS contractions were reduced by ACEA and SER-601, CB_1_ and CB_2_ receptor agonists, respectively, these effects being reversed in the presence of the corresponding cannabinoid receptor antagonists. (iv) In OZR bladder, the inhibitory action of ACEA on DSM nerve-evoked contractions were reduced versus those obtained in bladder from LZR, whereas that SER-601 failed to modify EFS responses in OZR.

Both the MS and IIDM are known risk factors for LUTS [2]. LUTS includes at least 3 or 4 clinical signs, such as, nocturia, incomplete emptying of the bladder and hesitant and weak urinary stream [2,16]. Epidemiological studies show a close relationship between the feature hyperinsulinemia cardiovascular disease, diabetes, obesity and risk factors triggers of MS with the development of LUTS in elderly men 4]. Indeed, vascular risk factors as obesity, diabetes, atherosclerosis, dyslipidemia, hyperglycemia and hypertension are related to the presence of LUTS [17].

With aging the risk of diabetes and cardiovascular disease increases significantly with episodes of UI associated with obesity. Thus, obese individuals of both sexes have an increased incidence of urgency UI episodes [3–6] and urodynamic studies show that patients with IIDM have increased urinary flow and residual volumes decreased [18]. In MS and IIDM there are characteristics low levels of high density lipoprotein, high triglyceride levels, atherosclerosis and hypercholesterolemia with hypertension combined with hyperlipidemia, which are risk factors for the UI in animal models and humans [19–21]. Diet-induced obesity models show an increased expression of M_2_ and M_3_ muscarinic and P2X_3_ purinergic receptors in the urothelium and the smooth muscle, as well as of the transient receptor potential vanilloid 1 channel, purinergic P2X_3_ receptors and inducible NO synthase proteins in the urothelium. All these protein expression changes have been related with the sensory dysfunction of bladder mucosa and LUTS in rats with MS [5,22,23,24].

Endocannabinoid system plays a key role in regulation of bladder function under physiological and pathophysiological conditions [7–10]. CB_1_ and CB_2_ receptor expression was previously confirmed in rat and human detrusor and urothelium [7,25,26]. This together with co-expression of vesicular ACh transporter and CB_2_ receptor and the inhibitory effect of CB_1_ and CB_2_ agonists on bladder neuronal contractions suggest a modulatory role of cannabinoid receptors on cholinergic nerve activity [7,26,27,28]. Cannabinoid receptor activation also exerts a direct effect on mechanosensory afferent function [8,23,24,25,26,27]. In fact, CB_1_ and CB_2_ receptors are involved in the reduction of the Aδ and C fiber activities in primary afferents of rat bladder produced by blockade of peripheral fatty acid amide hydrolase [8]. In the current study, the labeling with the neuronal marker PGP 9.5 showed a decreased bladder nerve fiber density. These data agree with those found in DSM from fructose-fed rats, where a diminished intrinsic innervation density of bladder wall was observed [24].

Hyperlipidemia is associated with increased urinary frequency and decreased bladder blood vessel and nerve density in rats [21]. MS with hyperlipidemia and hyperglycemia impair mitochondrial structure and function increasing the expression of NADPH oxidase and reactive oxygen species (ROS) [29]. An increased oxidative stress and bladder ischemia cause bladder nerve dysfunction, nerve fiber injury, mitochondrial injury and detrusor muscle cell damage [30], so that this pathogenic mechanism might be involved in bladder neuropathy from OZR. Further studies will be necessary to assess this possibility.

In OZR bladder, immunohistochemistry and western blot assays, using CB_1_ and CB_2_ selective antibodies, also showed a marked reduction in the immunoreactivity and CB_1_ and CB_2_ cannabinoid receptor protein expression in smooth muscle layer. Moreover, colocalization studies, by using cannabinoid receptor antibodies together with PGP 9.5, showed a reduced expression of CB_1_ and CB_2_ receptors located within nerve fibers distributed in the DSM from OZR versus with that exhibited in LZR. These results agree with those obtained in detrusor of patients with bladder overactivity, where cannabinoid CB_1_ and CB_2_ receptor expression in cholinergic nerves is reduced, thus indicating a role of cannabinoid receptors in the pathophysiology of overactive bladder [7].

Diabetic rats by high fructose consumption or administration of streptozocin have an increase in weight, capacity and bladder compliance and consume and excrete higher volumes of water compared with controls and exhibit hypertrophy, fibrosis, reduced total content of collagen and vascular edema of the bladder wall [3,12,31]. These animals show an altered cystometry characterized by increased phasic contractions, whereas the duration and amplitude of voiding contractions, as well as the content of ATP and ACh in bladder smooth muscle are reduced. This, together with the deterioration of the external urethral sphincter-electromyogram activity are proposed as causing of MS-related LUTS [3,24,32]. In the current study, the fact that contractions induced by KPSS, ACh, CCh and α,β-met-ATP were similar in LZR and OZR bladders is indicative of an intact myogenic contractility in OZR. These results contrast with those obtained in fructose fed obese rats, where contractile responses to carbachol were decreased significantly, responsiveness to high concentrations of ATP was increased and M_2_,M_3_-muscarinic and P2X_1_ receptor protein expression in the urothelium and muscle layer was significantly increased [3,5,22,24]. Our data also differ with those obtained in DSM from Zucker diabetic fatty rats at 27 weeks, where carbachol contractile responses were increased possibly as a consequence of the compensated state of diabetic bladder dysfunction [33].

In our study, atropine-sensitive and -resistant nerve-evoked contractions were reduced in OZR DSM, which may be explained by the diminished nerve fiber density observed. These findings agree with those obtained in the fructose-induced obese rat bladder, where neurogenic contractions were significantly reduced [3,24]. The fact that the excitatory neurotransmission of the bladder was impaired in both obesity models, genetic and induced by diet, suggests a key role for neuropathy in MS-associated bladder dysfunction. In the current work, CB_1_ and CB_2_ receptor agonists and antagonists failed to modify the DSM tension from LZR and OZR, thus suggesting that postjunctional cannabinoid receptors seem do not play a significant role on DSM myogenic contractility. In LZR bladder, however, selective activation of CB_1_ and CB_2_ cannabinoid receptors inhibited the nerve-evoked contractions. These results agree with those previously described concerning the modulatory role of cannabinoid receptors on cholinergic nerve activity [25,26,28]. In OZR bladder, the reduced expression of CB_1_ and CB_2_ receptors located within nerve fibers and nerve density in the DSM from OZR, as well as the reduced inhibition promoted by the selective activation of CB_1_ receptors and the lack of effect of CB_2_ receptor stimulation on EFS responses suggest an altered modulation of prejunctional inhibitory cannabinoid receptors on nerve-evoked contractions, probably related with a reducing ACh and ATP release in the OZR bladder. The down-regulation of both prejunctional CB_1_ and CB_2_ cannabinoid receptors in OZR bladder probably might be ascribed with the MS-related neuropathy. Neuronal cannabinoid receptor activation reduces the contraction induced by transmitters released from parasympathetic nerves in DSM [25,26,28]. Thus, one would expect to obtain a higher nerve-evoked contraction of bladder from OZR. The fact that this response was reduced is explained by the diminished nerve fiber density showed in OZR bladder. These results are consistent with those obtained in a cannabinoid CB_1_ receptor knockout (KO) mouse, which exhibits a higher micturition frequency and increased bladder spontaneous activity. In the KO mice model, carbachol-induced myogenic contractions were similar to that obtained in controls, but EFS-elicited neurogenic responses were significantly diminished [34].

A pharmacological modulation of the endocannabinoid system has been proposed to be beneficial for widespread diseases of urinary bladder, such as cystitis and hyperactive bladder. In fact, drugs that inhibit endocannabinoid degradation and raise the level of endocannabinoids co-released with the endocannabinoid anandamide, have been proposed as useful for bladder dysfunction [9,10]. In the current study, the fact that a down-regulation of CB_1_ and CB_2_ cannabinoid receptors is involved in the impaired nerve-evoked contractions from OZR bladder, reinforces the role of endocannabinoid system as an interesting and promising therapeutic target for MS-related bladder dysfunction. In conclusion, these results suggest that a diminished function and expression of neuronal cannabinoid CB_1_ and CB_2_ receptors, as well as a lower nerve fiber density is involved in the impaired excitatory neurotransmission of the urinary bladder from the OZR.
